# Enhancing surface detection: A comprehensive analysis of various YOLO models

**DOI:** 10.1016/j.heliyon.2025.e42433

**Published:** 2025-02-04

**Authors:** G. Deepti Raj, B. Prabadevi

**Affiliations:** School of Computer Science Engineering and Information Systems, Vellore Institute of Technology University, Vellore, 632014, Tamilnadu, India

**Keywords:** Convolution neural networks, YOLOv5, Attention mechanisms, Object detection, Defect detection, LeakyReLU

## Abstract

Material defects can significantly affect strength, durability and overall quality. Complex backgrounds and variations in steel surface images often hinder productivity and quality in industrial environments. Accurate defect detection becomes difficult due to small target size and unclear features. However, implementing accurate and automated object detection algorithms mitigates these challenges, allowing errors or defects to be detected before processing. Version 5 of You Only Look Once (YOLO), a precisely optimized learning model, has undergone extensive testing on steel strip datasets, providing effective solutions for recognition and detection in industry environments. This study presents an improved YOLOv5 detection model, exploiting the efficient channel attention (ECA) and coordinated attention (CoordAtt) mechanisms. Our results show notable improvements, with the ECA hybrid attention mechanism achieving 2–4 times faster inference times while maintaining high accuracy. Additionally, CoordAtt integration minimizes the parameter count by 25 % and gives higher accuracy on one of the datasets. Comparative analysis with YOLOv6, YOLOv7, and YOLOv8 demonstrates the superior accuracy of the enhanced YOLOv5 model on the NEU-DET and GC10-DET steel strip benchmark datasets, highlighting its effectiveness in detecting and timely recognition of actual defects.

## 1 Introduction

Deep learning (DL) techniques are transforming object detection by learning object characteristics directly from data, paving a way for significant enhancements in the field. The impact of small and dense defects vary between different types of steel strip, each with its own characteristics and defect size. Traditional manual testing methods are plagued by low detection rates, time-consuming processes, and prone to false positives. Although image processing techniques such as Local Binary Pattern (LBP) [1], Histogram Oriented Gradient (HOG) [2], Gray-Level Co-Occurrence Matrix (GLCM) [3], Support Vector Machine (SVM) and Nearest Neighbor Classifier (NNC) have enhanced surface defect detection, their dependence on fine-tuned threshold parameters limits their adaptability to changing environmental conditions. As a result, the need for accurate and automated object detection algorithms remains critical in industries focused on continuous product quality improvement and efficient delivery processes.

### 1.1 Object detection

Since Krizhevsky's groundbreaking development of AlexNet in image classification in 2012, deep neural networks have become the focal point of object recognition research [4,5]. The integration of image processing techniques with artificial intelligence is progressing rapidly, supporting numerous real-time applications across various industries, including agriculture, manufacturing, healthcare and more [[6], [7], [8], [9]]. Object recognition and detection play a vital role in industries like autonomous vehicles, surveillance systems, retail, robotics, and medical imaging, enabling applications such as player tracking, wildlife monitoring, inventory management, anomaly detection, and error/defect identification.

Recently, DL techniques have made significant breakthroughs in industrial fault/defects diagnosis, offering opportunities to reduce labor costs while enhancing the speed and accuracy of detection. Modern object detection methods, including two-step and one-step methods, have attracted substantial attention. For example, Deepak Kumar et al. uses a fully convolutional Siamese network (s-FCN-loc) to accurately segment road segments for smart vehicle systems [10]. Similarly, the hybrid Flight Moth Search algorithm, which uses a two-stage deep convolutional neural network (DCNN) to classify roads and lanes [11]. These DL models demonstrate strong feature representation, with two-stage algorithms like R-CNN (region-based convolutional neural networks) excelling in detection accuracy but often lack in real-time performance [12]. In contrast, one-step detection algorithms such as YOLO prioritize high detection rates, making them widely applicable across various domains [[13], [14], [15], [16]].

### 1.2 Defect detection

In the steel industry, many factors can hinder the production. Steel strips serve as a key raw material for industries like manufacturing, aerospace and electronics, require high surface quality. Although steel belts possess excellent properties such as corrosion resistance, wear resistance and fatigue resistance, they are susceptible to defects, which can result in losses and pose potential safety risks. Defects on the steel surface, often characterized by a gray or white color, uneven distribution and a variety of sizes and shapes, require a meticulous defect detection process to meet the product quality standards. A wide variety of defects including inclusions, wrinkles, water spots, silk stains, edge cracks, burned edges, pores, mill scales, pitting, stains, capillary cracks, breaks and marks scratches may appear on the steel surface. This study explores the use of machine vision and computer vision techniques to detect surface defects on steel strips during processing.

### 1.3 Literature survey

#### 1.3.1 Traditional methods of steel strip defect detection

As artificial intelligence moves to mobile devices, smaller DL models are needed to improve speed and reduce battery consumption. Chen. K. et al. [17] proposed a fast Rank SVM with kernel approximation to address long training times and computational costs. CNN-based architectures are popular for visual defect detection and automatic feature extraction. Cha and Choi [18] proposed a DCNN for crack detection on steel and concrete surfaces, showcasing its robustness in various real-life scenarios. Additionally, to enable near real-time detection of variousdefect types simultaneously, they developed a structural visual inspection method utilizing the Faster Region-Convolutional Neural Network (Faster R-CNN) [19].

Lee et al. [20] used the YOLO variant to detect surface defects of flat steel, achieving a 99 % detection rate on the cold-rolled steel surface. Zhao et al. [21] used a combination of Generative Adversarial Networks (GAN), Auto Encoder (AE), and LBP to identify defects on steel structure surfaces without manual labelling, demonstrating competitive performance. Mei et al. [22] used reconstructed residual maps and a convolutional AE network to reduce noise, thus achieving successful recognition without human involvement.

Youkachen et al. [23] innovatively utilized Convolutional AE (CAE) to reconstruct images with defects and highlight shape features, demonstrating the versatility of unsupervised learning methods. Ren et al. [24] proposed an automatic inspection technique for surfaces with defects, reducing the defect avoidance rate compared to the baseline. Zhou et al. [25] developed a new bi-linear model designed to extract both global and local features of surface defects, allowing weakly supervised defect identification and classification. Furthermore, He et al. [26] presented CAE-SGAN, a new technology that combines semi-supervised GAN (SGAN) and CAE to localize detection regions.

#### 1.3.2 YOLO-based defect detection

Object recognition algorithms play a central role in various fields, from medicine to transportation, and drive cutting-edge technologies. Historically, object detection involved complex multi-stage pipelines like, R-CNN and fast R-CNN, which were time-consuming and unsuitable for real-time implementation. In contrast, YOLO uses a single neural network for all elements of the task, speeding up the process (45 fps) making it faster and easier to optimize.

Joseph R et al. introduced YOLO in 2015. YOLO divides 2D images into a grid, with each cell tasked with detecting objects within a designated area. The network adjusts the overall image properties and independently predicts the bounding boxes for each cell, enabling real-time object detection with improved detection rate and accuracy. The term “YOLO” symbolizes living in the present moment without worrying about the future. YOLO makes predictions for training and validation images in a single algorithm run, eliminating background noise [39]. Table 1 contains an analysis of the YOLO-based surface defect detection method for steel strips.

**Table 1:** Analysis of YOLO based surface defect detection methods for steel strip.

Ref	Name of the different YOLO-based Models	Description
[27]	Channel attention, and bidirectional feature fusion in a fully convolutional one-stage (CABF-FCOS)	Improves the defect detection performance by reducing feature information loss and performing feature fusion.
[28]	Deformable convolution enhanced backbone network	The detector network locates and categorizes steel surface defects by extracting complicated information from multi-shape steel surface defects.
[29]	Classification priority YOLOv3 DenseNet) neural network	Priority classification was implemented on the images by the YOLOv3 basic network. As a result, the network processes multi-layer convolution features generated by the dense connection block before making predictions, enhancing feature reuse and fusion.
[30]	Improved YOLOv4Algorithm	The path aggregation network is reconstructed into a customized receptive field block structure with the attention mechanism incorporated into the backbone network, enhancing the model's feature extraction capability.
[31]	Optimized YOLOv3 Algorithm	Neural convolutions, batch normalization (BN), and leaky Rectified Linear Unit (RELU) activation are used to create 4 residual layers (res units), which optimize the network's capacity for feature extraction and learning while preventing gradient vanishing.
[32]	Reconstructed image-based detection method and compressed representation-based detection method	CAE is used as the compression pre-processing framework for detection, with the YOLO-tiny network serving as the backbone for the detection framework.
[33]	Improved PP-YOLOE-m Detection Network	Spatial Pyramid Pooling (SPP) in the neck network expands the receptive field for better global information, while data augmentation and CoordAtt enhance spatial location awareness.
[34]	Improved YOLOv3	Data Augmentation is used to increase robustness of the algorithm and the K-means++ technique, which has less randomness, was utilized to do clustering analysis on defect labels with the appropriate selection of anchor boxes.
[35]	Improved YOLO Model with all Convolutional	An all-encompassing solution for the detection is provided by 27 convolutional layers and data augmentation.
[36]	YOLO-V3-based end-to-end defect detection model	The anchor-free feature selection approach with an anchor-based structure reduces the computation time. Custom dense convolution blocks are employed to extract rich feature information, enhancing the network's ability to characterize.
[37]	YOLOv5 with Attention Mechanisms	The K-means algorithm clusters the defect samples and incorporates channel attention and spatial attention mechanisms into the feature extraction network.
[38]	Improved YOLOv5 with ShuffleNetV2	A CoordAtt mechanism is used to reduce the feature information loss, and lightweight ShuffleNetV2 is used to improve the real time performance of defect detection.

Notably, YOLO-based surface defect detection for steel strip shows high accuracy and mean average precision (mAP) compared to other real-time systems. YOLO offers easy deployment, low complexity, and flexibility for images and videos.

Successive versions of YOLO, including YOLOv2 [40], YOLOv3 [41], YOLOv4 [42] and YOLOv5 [[43], [44], [45]] have introduced improvements such as: YOLOv2 integrates distance indicators, IOU, and batch normalization across all convolutional layers to improve recall and precision. YOLOv3 uses Residual Network (ResNet) [46] and Darknet-53 to improve image recognition and reduce false background detection with Feature Pyramid Network (FPN) [47]. YOLOv4 introduces feature extraction and replaces cross-stage partial Darknet-53 with Darknet-53, and integrates the Path Aggregation Network (PANet) [48] and (SPP) [49] modules to support instance segmentation. These advancements have enhanced YOLO's performance and versatility.

### 1.4 Research motivation

The YOLOv5 model offers significantly improved performance in detecting smaller objects while operating 2.5 times faster than its predecessors. This is achieved by processing the entire image through a single neural network, dividing it into sections to predict the bounding boxes. Despite challenges in deploying large models on devices with limited resources due to increasing space and power requirements, Ultralytics has effectively used the open source YOLOv5 model for training and inference. Our contributions include.

**Fig. 1:**
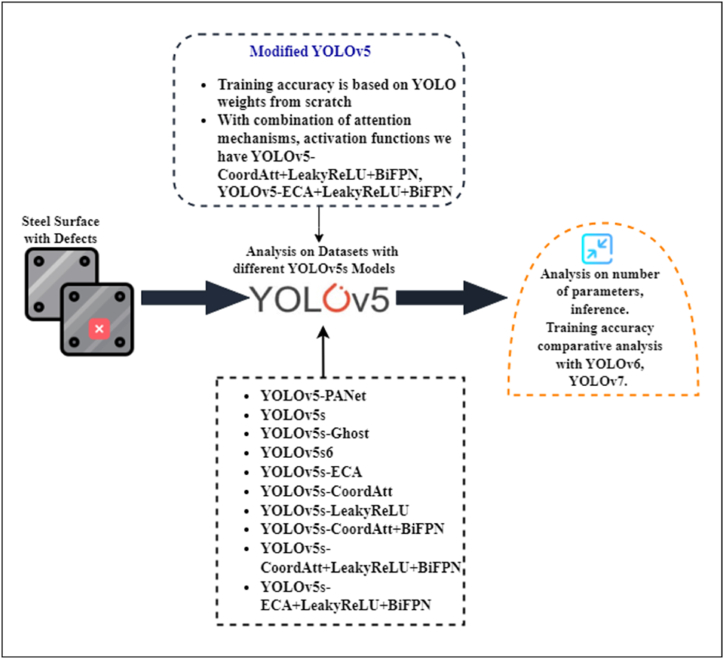
Problem description.

**Fig. 2:**
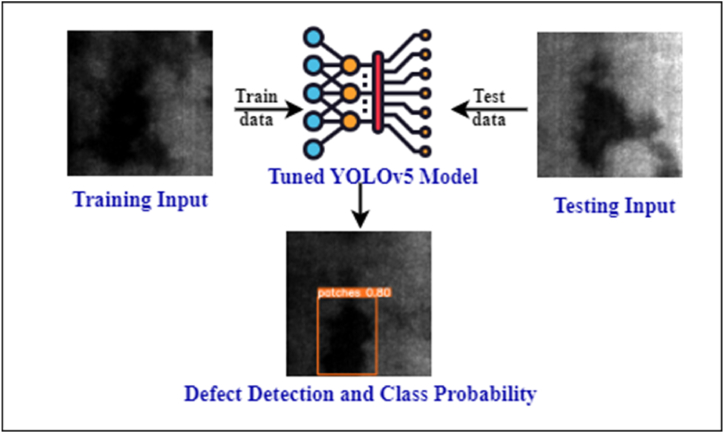
Flowchart of the experiment.

1. Selecting YOLOv5 as the base model and evaluating its accuracy, inference speed, and number of parameters. We trained the steel strip datasets using YOLOv5 PANet, and compared it with YOLOv5s6 and the light-weight YOLOv5s-Ghost model.
2. Implementing the ECA attention mechanism which enhances beneficial features and eliminate unwanted ones, resulting in a slight increase in recognition speed.
3. Integrating CoordAtt to improve object representation in deep neural networks by considering channel interactions and position information, leading to parameter reduction and improved model performance.
4. Leaky Rectified Linear Unit (LeakyReLU) is used as the activation function, with BiFPN combining features across multiple scales for efficient merging.
5. A comparative analysis of various models, including YOLOv5s-PAnet, YOLOv5s6, YOLOv5s-ECA, YOLOv5s-CoordAtt, YOLOv5-LeakyReLU, YOLOv5s-BiFPN and their combinations is conducted.
6. Our experiments, comparing YOLOv5 with YOLOv6, YOLOv7, and YOLOv8, highlight the superior performance of the YOLOv5 hybrid model. Models with ECA attention mechanism notably show shorter inference times and fewer parameters. Fig. 1, Fig. 2 depict the problem definition and experimental flowchart, respectively.

The rest of this paper is divided into the following sections. Materials and Methods discussing YOLOv5 and the integration of ECA and CoordAtt were discussed in Section 2, followed by experiments in Section 3 which describes datasets, evaluation specifications, and metrics, Section 4 covers results and discussions, and finally the paper is concluded in Section 5.

## 2 Materials and methods

In this study, we use the YOLOv5s model as the benchmark on the NEU-DET and GC10-DET datasets.

### 2.1 Overview of YOLOv5

YOLOv5, proposed by Glenn Gocher in 2020, integrates advanced optimization techniques into the YOLO object detection architecture. Despite the lack of official publications, YOLOv5 has been widely adopted and continues to receive updates, indicating its importance [50]. YOLOv5 is 90 % smaller than YOLOv4 and outperforms its predecessors in speed. Leveraging the PyTorch framework, YOLOv5 provides seamless migration to Open Neural Network Exchange (ONNX) and CoreML, facilitating deployment on embedded and mobile devices. The inclusion of Bottleneck Cross Stage Partial (CSP) modules with varying depths and widths on YOLOv5 models (YOLOv5s, YOLOv5m, YOLOv5l, YOLOv5x, YOLOv5n), enhances their adaptability. Initially trained on the COCO dataset, which includes 200,000 labeled images across 80 classes, the YOLOv5 network architecture consists of four layers: Input, Backbone, Neck, and Head.

(1) **INPUT:** The input layer consists of 3 modules.
● *Mosaic data enhancement:* YOLOv5's input layer employs a mosaic data augmentation method to enhance detection accuracy.
● *Adaptive anchor frame calculation:* YOLOv5 adaptively calculates the anchor frame, determining the optical anchor box value during each training phase.
● *Adaptive image scaling:* The original image is re-sized to fit the default size of the recognition network.
(2) **BACKBONE:** The YOLOv5 Backbone Network integrates focal structures and CSPNet structures for convolution and pooling, extracting important information from image samples [51]. It includes various modules such as focus module, CBL module (Conv-BN-LeakyReLU), CSP1-X module, BottleneckCSP module for channel limiting and expansion as well as SPP module. The BottleneckCSP module combines the approach of Jianwiyo Wang [52] with Kaiming's SPP module from Bottleneck [53].
● Focus structure: This structure initially divides the 416 × 416 × 3 input image into a 208 × 208 × 12 image, then creates a feature map using convolution with 64 kernels. Subsequent convolution, clustering, and down-sampling operations extract image features at horizontal and vertical intervals.
● CSP architecture: CSPNet solves the multi-gradient problem, by reducing floating-point operations (FLOPS) and algorithm parameters. It divides the base class into two parts, merging them with a multi-level hierarchy to reduce computational complexity and improve accuracy. In YOLOv5, CSP2_X is applied to the neck and CSP1_X is applied to the backbone network.
(3) **NECK:** The Updated YOLOv5 neck layer applies FPN-PANet structure, improving low-level features. FPN facilitates top-down semantic feature collection, while PANet is a bottom-up bidirectional enhancement framework. The ConcatFun block facilitates tensor chaining operations, thus minimizing information loss and gaining more fundamental information. The backbone of YOLOv5 acts as a feature extractor, with the head locating bounding boxes to detect objects in each frame.

**Fig. 3:**
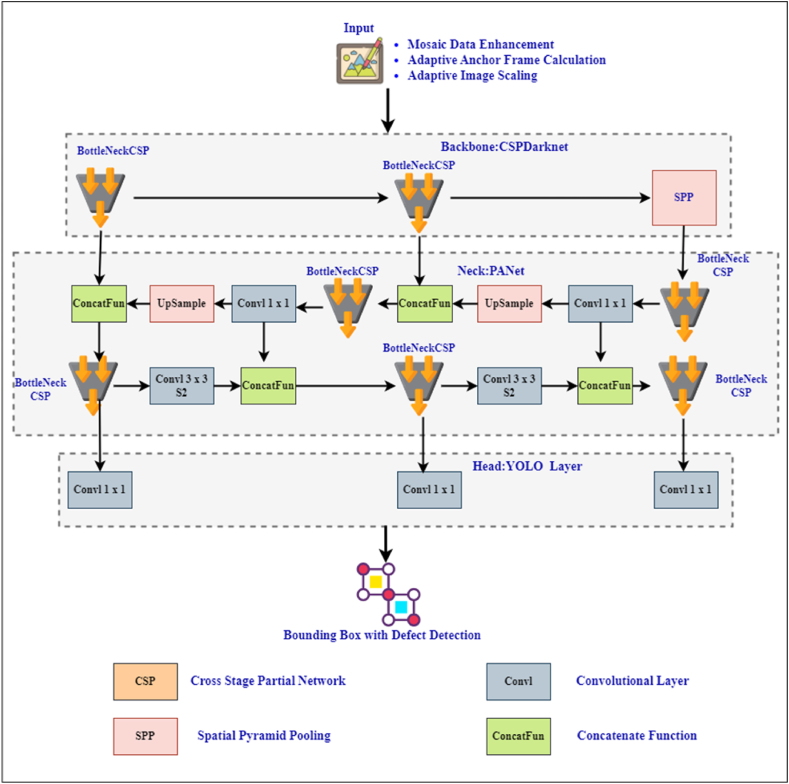
YOLOv5 architecture.

The bounding box position is calculated as shown in equation (1).

(1) $$\sigma_{r}^{g}=O_{r,g}*{℧}_{T}^{P}$$

where r is the bounding box of the grid g, and $\sigma_{r}^{g}$ is the confidence score of the r bounding box. r and g represent the target, and if the target is within the g box, the value of $O_{i,j}$ would be 0; otherwise, it would be 1. $O_{i,j}$ is the intersection over union(IOU), a fairly well-known evaluation metric in image detection. The IOU score is calculated on how precisely the bounding box around the target is placed. Fig. 3 details the original YOLOv5 architecture, showing all layers including ConcatFun, Convl, BottleNeckCSP, and UpSample blocks.

(4) **HEAD:** The head is responsible for the final detection process, predicting classes, image features, and creating bounding boxes around the target elements. It uses classification loss and bounding box regression loss as loss functions for target identification. YOLOv5 handles non-overlapping bounding boxes by employing Generalized Intersection over Union (GIOU) as the loss function and applying non-maximum suppression to select the optimal bounding box.

### 2.2 YOLOv5s-ghost

This module compresses network parameters, reducing operating costs. By extracting spatial feature information at various scales, the model's robustness in spatial localization and object detection is enhanced [54]. In YOLOv5, Convl and C3 in the backbone and head layers are replaced with GhostConv and C3Ghost, reducing the learned parameters and minimizing overfitting. GhostConv allows for redundant data interpretation, facilitating better understanding of input data and compressing the depth and width of the model, saving computational costs.

### 2.3 YOLOv5s6

YOLOv5s6 represents the final version of 5th Edition, featuring significant changes to the backbone network layer including integrating the Spatial Pyramid Pooling Fast (SPPF) layer, an SPP variant, CBS (Conv BN SiLU), and CSP1 X (where X represents the ResUnit number). The neck layer retains the PANet FPN incorporating some standard convolutional layers and CSP2X (where 2X means the number of CBS). In addition, the sigmoid linear unit (SiLU), also known as the sigmoid weighted linear unit, serves as the activation function, enhancing the model's expressive ability by multiplying the sigmoid function with its input.

### 2.4 YOLOv5-PANet

Incorporating the independently developed YOLOv5 PANet structure, the model's neck layer improves instance segmentation by tracking spatial information. PANet ensures good retention of spatial information, facilitating accurate pixel positioning for mask training. Key features of PANet include bottom-up path scaling, adaptive feature clustering, and fully connected fusion, which promotes efficient information propagation throughout the process. Incorporating extension paths improves the functionality of each layer, making PANet simple, fast, and very efficient.

### 2.5 YOLOv5 -BiFPN

Replacing PANet at the neck layer, YOLOv5 BiFPN uses BiFPN, which has contrasting network weights and topologies to improve the balance of feature information across different scales. BiFPN enables fast and transparent multi-scale feature fusion by connecting input and output nodes within the same layer, improving unification and reduces information transmission time between upper and lower levels.

### 2.6 YOLOv5 -LeakyReLU

The LeakyReLU activation function determines the neuron's importance in the prediction process. Unlike ReLU, LeakyReLU's gradient coefficient is predetermined and is not adjusted during training, thus preventing the gradient from disappearing. Unlike parametric ReLU, where the gradient of the negative input is a hyper-parameter, LeakyReLU defines this gradient as a fixed value, making it unlearnable. LeakyReLU maintains continuity in its domain like ReLU, but it is not zero differentiable.

The formula for the LeakyReLU activation function is given by Equation (2) below.

(2) f(x)=max(0.01∗x,x)

Given a positive input, this function returns x. However, for a negative value, a very small value equal to 0.01 times x is returned, ensuring a non-zero slope on the left side of the graph, thus avoiding dead neurons. Although YOLOv5 uses the SiLU activation function by default, LeakyReLU is used in this work to maintain model sparsity and speed up training.

### 2.7 YOLOv5s-CoordAtt

In Machine Learning (ML), attention mechanisms play an important role in data processing, allowing models to focus on relevant information while ignoring irrelevant data. This concept forms the basis of the attention mechanism in computer vision. Recently, non-local neural networks [55] and self-attention mechanisms [56] have gained popularity for their ability to create spatial or channel-specific attention.

However, self-attention modules require significant computational resources, making them more suitable for large-scale models than cellular networks. CoordAtt addresses this challenge by capturing essential visual information. By integrating location information into the attention channel, CoordAtt improves the representation of objects of interest in deep neural networks. Unlike the Squeeze and excitation (SE) network [57], which includes compression and excitation steps, CoordAtt goes beyond incorporating both position information and channel interactions. This involves adjusting information integration and attention, leading to a more complete understanding of visual data. Fig. 5 gives structure of CoordAtt.

### 2.8 YOLOv5s-ECA

**Fig. 4:**
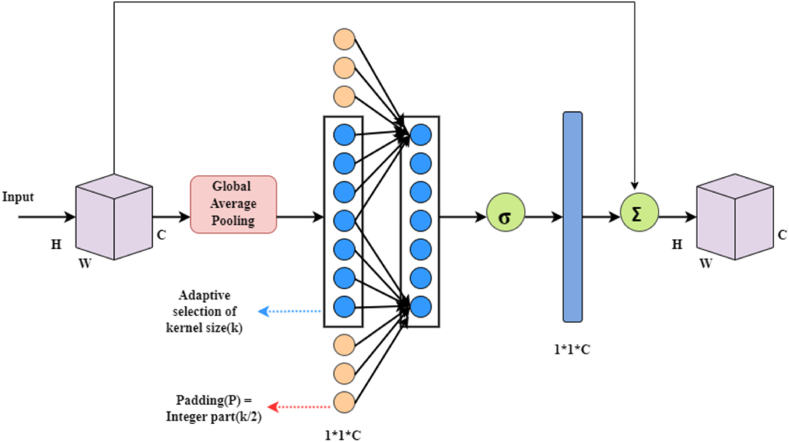
Structure of ECA

Attention mechanisms have demonstrated the potential to improve DCNN. The ECA module, introduced by Wang et al. [58], addresses the trade-off between performance and complexity by significantly improving performance with minimal parameters. ECA aims to develop efficient channel attention without overly complex models, allowing to capture cross-channel interactions without reducing dimensionality. ECA uses global average pooling (GAP) to generate channel weights from synthetic objects via fast one-dimensional (1D) convolution operations of size k, where k is a function of channel dimension C. Fig. 4 gives the structure of ECA.

**Fig. 5:**
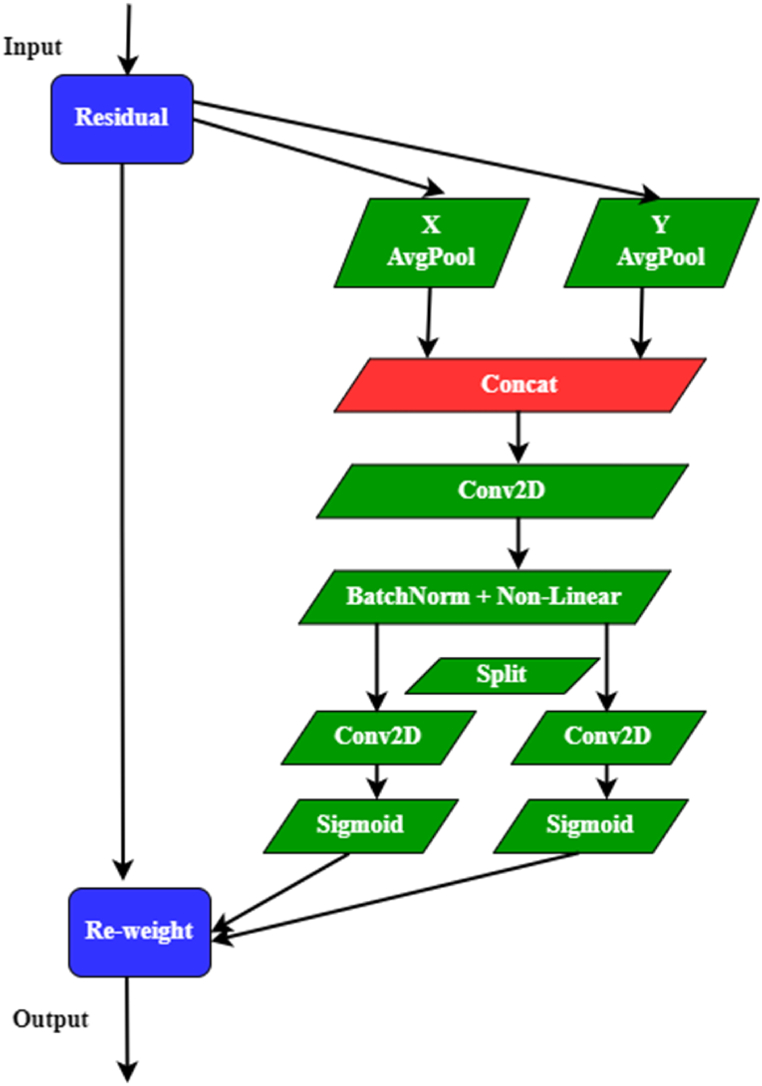
Structure of CoordAtt.

Mathematically, ECA can be expressed as equation (3), where k represents the kernel size, C1D represents the one-dimensional convolution, and σ represents the sigmoid function.

(3) $$\omega =\sigma \left(C1D_{k}\left(y\right)\right)$$

By enhancing useful features and eliminating unimportant ones, models can effectively discover, analyze, and predict new data in a timely manner. To integrate the ECA attention mechanism into the YOLOv5 architecture, the ECA module replaces the final C3 module [59] in the backbone.

The steps of the ECA Attention module are described below:

**Input:** Number of channels for input feature map and kernel size.

**Assumptions:** Apply Avgpool on input signal, Conv1D for convolution operation on first axis.

**Output:** Multi-scale information fusion.

1. Select kernel size (ksize).
2. Determine the number of channels (c) for the feature map based on ksize.
3. Run Conv1D using the ksize and padding values (ksize-1).
4. Get the spatial information feature descriptor of the input tensor.
5. Use PyTorch functions (Squeeze, Transpose Convolutions and Unsqueeze) to get a new tensor with all dimensions.
6. Predict large-scale information fusion using sigmoid probability.

**Fig. 6:**
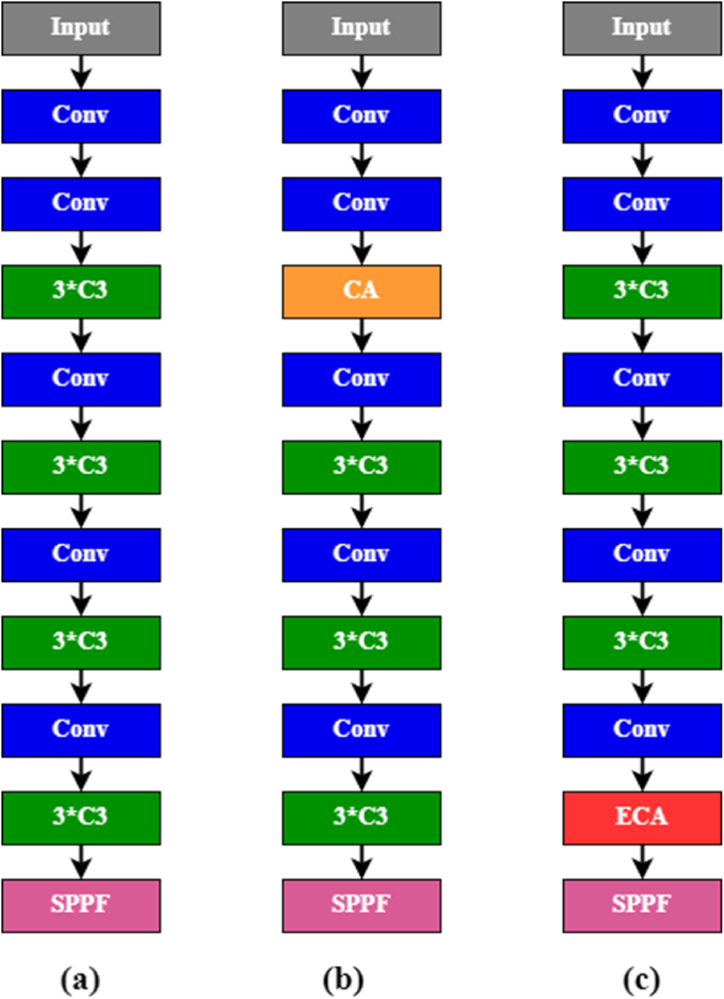
(a) YOLOv5 backbone (b) YOLOv5-CoordAtt (c) YOLOv5-ECA.

We modified the YOLOv5s model by integrating a combination of attention mechanisms, activation functions, and BiFPN, including YOLOv5s-CoordAtt, YOLOv5s-CoordAtt + BiFPN, YOLOv5s-CoordAtt + LeakyReLU + BiFPN, YOLOv5s-ECA. These modifications aim to evaluate training accuracy, inference time and number of parameters on the dataset. Fig. 6(a) represents the original YOLOv5 backbone, Fig. 6(b) and Fig. (c) denotes the place where CA and ECA modules are added to the backbone of YOLOv5s.

## 3 Experiments

### 3.1 Datasets description and experimental setup

Our study uses two main data sets for steel strip surface defects: NEU-DET and GC10-DET. The NEU-DET dataset is sourced from Northeastern University and the GC10-DET dataset is collected from industrial manufacturing facilities. Both datasets were labeled and annotated using ROBOFLOW and split into 70 % for training, 20 % for validation, and 10 % for testing. The types of steel strip surface defects in the NEU-DET and GC10-DET datasets are detailed in Table 2.

**Table 2:** Defect types description.

S.No	Defect Types	Description
1.	Patches	Patches occurs because of heat treatment, corrosion
2.	Pittedsurface	Pitting corrosion, also known as pitted surface, is a very localized type of corrosion that results in the random formation of tiny holes on the steel surface.
3.	Crazing	A network of small fractures on a material's surface is referred to as crazing.
4.	Rolled-in scale	A rolled-in scale defect occurs when mill scale, a flaky iron oxide that forms on heated steel, gets rolled into the metal during the rolling process.
5.	Inclusion	A type of steel imperfection called an inclusion has a significant impact on the material's polishability, ductility, and fatigue strength. During steel treatments in the ladle, inclusions are created.
6.	Scratches	This defect is caused by a foreign object that is present in a rolling mill, pickle line, pass line etc.
7.	Water Spot	Water spot on steel strip surfaces are a result of the immersion lithography process.
8.	Oil Spot	The high mixed oil content of the emulsion that was left on the strip surface gives rise to the rolling oil spot.
9.	Punching hole	Blind holes, irregular distances, connected holes may emerge during the punching process on surfaces.
10.	Silk Spot	A silk spot is a wave-like plaque that develops on the surface and is frequently caused by an uneven roller temperature or pressure.
11.	Weld line	Incorrect welding locations or improper welding are the causes of the weld metal defect.
12.	Crease	During the uncoiling process, a metal strip might develop vertical or transverse folds.
13.	Crescent Gap	A half-circle-shaped defect caused by cutting.
14.	Waist Folding	Folds that look like wrinkles due to low-carbon
15.	Rolled Pit	Periodic pits or bulges on the metal's surface, usually caused by tension roll damage.

The NEU-DET dataset comprises of 1440 grayscale images, each with a resolution of 200∗200 pixels with six surface defect types: patches(Pts), pitted surface (Pse), crazing (Cg), rolled-in scales (Ris), inclusions (Iln) and scratches (Sts).

**Fig. 7:**
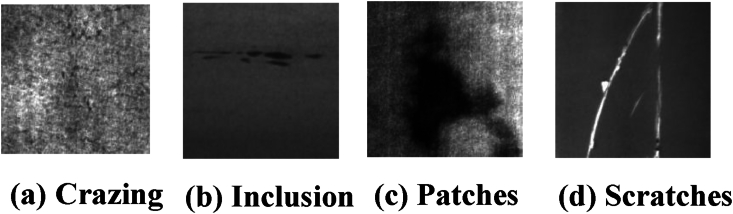
Sample images from NEU-DET Dataset.

Fig. 7(a), (b), 7(c) and 7(d) denotes crazing, inclusion, patches and scratches which are some of the sample images, while Table 3 illustrates the distribution of samples in the NEU-DET dataset.

**Table 3:** Sample distribution of NEU-DET dataset.

Defect Category	Cg	Iln	Pts	Pse	Ris	Sts	Total
Number of Images	240	240	240	240	240	240	1440

GC10-DET contains 2312 grayscale images with a resolution of 2048 ∗ 1000 pixels with ten surface defect types: water spots (Wrt), oil spots (Ot), punching holes (Png), silk spot (Sst), inclusion (Iln), weld line (Wll), crease (Cre), crescent gap (Cgp), waist folds (Wfg) and rolled pit (Rpt). The dataset labels the defects as follows: 1_chongkong for Png, 2_hanfeng for Wll, 3_yueyawan for Cgp, 4_shuiban for Wrt, 5_youban for Ot, 6_siban for Sst, 7_yiwu for Iln, 8_yahen for Rpt, 9_zhehen for Cre and 10_yaozhe for Wfg.

**Fig. 8:**
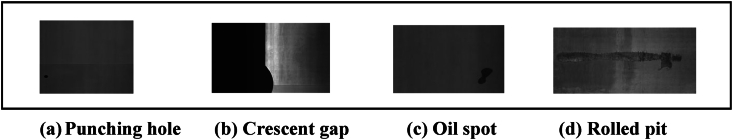
Sample images from GC10-DET Dataset.

Fig. 8(a) and (b), 8(c) and 8(d) denotes punching hole, crescent gap, oil spot, rolled pit which are some of the sample images, while Table 4 shows the distribution of samples in the GC10-DET data set.

**Table 4:** Sample distribution of GC10-DET dataset.

Defect Category	Png	Wll	Cgp	Wrt	Ot	Sst	Iln	Rpt	Cre	Wfg	Total
Number of Images	219	273	226	289	204	651	216	31	53	150	2312

### 3.2 Evaluation specifications and metrics

Our study evaluates model performance using the metrics mean average precision (mAP50), precision (P), and recall (R). R means the percentage of surface errors that are successfully identified, while P evaluates the accuracy of error predictions. mAP serves as a measure for object detection accuracy. Frames per second (FPS) measures the model's real-time processing speed, estimated using equation (4).

(4) $$\text{FPS}=\frac{\text{No}\;\text{of}\;\text{frames}}{\text{Total}\;\text{detection}\;\text{time}\;\left(s\right)}$$

Higher Precision, recall, and FPS indicate better model performance, with faster and more accurate object detection capabilities.

### 3.3 Experimental hardware configuration and set-up

Our experiments utilizes a Python implementation of YOLOv5, available on GitHub. The processing platform is a Windows 10 desktop, with 12 GB memory and an Intel(R) Core(TM) i5-1035G processor. For deployment, we chose Google Colaboratory Notebook, leveraging NVIDIA Tesla T4 GPU, Python 3.7.13 and Torch Framework 1.11.0 cu113.

### 3.4 Parameter composition and training

The performance of the enhanced YOLOv5 network is evaluated using key evaluation metrics. To optimize the processing results, the input image size is normalized to 416 × 416 for training and validation. The re-sized images still maintain the essential features and defects present in the original images [60]. The batch size is set to 16 and training epochs are set to 300 for improved efficiency. Scratch weights are used for training. The optimization function used is SGD with weight decay 0.0005, a momentum of 0.937, and an initial learning rate of 0.01 are used to speed up model convergence. The trained weights are recorded for the best epoch and the last epoch.

## 4 Results and discussions

This study explores and analyzes various underlying models of YOLOv5, including YOLOv5s, YOLOv5s-Ghost, YOLOv5s6, YOLOv5s-PAnet, and the combination of attention mechanisms and activation functions such as YOLOv5s-LeakyReLU, YOLOv5s-ECA, YOLOv5s -CoordAtt, YOLOv5s-CoordAtt + BiFPN, YOLOv5s-CoordAtt + LeakyReLU + BiFPN and YOLOv5s-ECA + LeakyReLU + BiFPN.

We also compare these models with recently releasedYOLOv6, YOLOv7, and YOLOv8. The training uses scratch weights to ensure realistic results. Table 5 shows the training results of the YOLOv5s model on the NEU-DET dataset, achieving a total mAP of 0.613, with the class of patch defect achieving a 0.895 mAP. In addition, Table 6 shows the training results of the YOLOv5s model on the GC10-DET dataset, achieving a total mAP of 0.676, with a classification result of 0.965 mAP for punching hole class defect.

**Table 5:** YOLOv5s training on NEU-DET

Classes	mAP@0.5 (%)	Recall (%)	Precision (%)
Pts	0.895	0.912	0.675
Pse	0.538	0.629	0.525
Cg	0.542	0.479	0.525
Ris	0.646	0.683	0.623
Iln	0.784	0.821	0.625
Sts	0.672	0.784	0.668

Overall	0.613	0.683	0.548

**Table 6:** YOLOv5s training on GC10-DET.

Classes	mAP@0.5 (%)	Recall (%)	Precision (%)
Wrt	0.695	0.76	0.684
Ot	0.61	0.678	0.501
Png	0.965	0.957	0.781
Sst	0.559	0.613	0.529
Iln	0.384	0.554	0.409
Wll	0.889	0.968	0.798
Cre	0.513	0.526	0.504
Cgp	0.909	0.903	0.662
Wfg	0.901	0.918	0.888
Rpt	0.209	0.231	0.254

Overall	0.676	0.685	0.637

Table 7 presents the results of analyzing the NEU-DET dataset using various YOLOv5 models. YOLOv5-PANet and YOLOv5s6 have more parameters and longer inference times compared to the other YOLOv5 models.

**Table 7:** Result analysis on NEU-DET dataset with different YOLOv5s Models.

Models	Neck-layer Structure	# Params.	Training mAP (%)	Inference (ms)
YOLOv5-PANet	PANet	46135203	0.563	28.4
YOLOv5s	FPN + PANet	7026307	**0.613**	8.1
YOLOv5s-Ghost	FPN + PANet	**3689211**	0.608	9.4
YOLOv5s6	FPN + PANet	12327460	0.568	10.6
YOLOv5s-ECA	FPN + PANet	7217414	0.601	**7.7**
YOLOv5s-CoordAtt	FPN + PANet	**5151875**	**0.618**	8.4
YOLOv5s-LeakyReLU	FPN + PANet	7026307	0.745	8.8
YOLOv5s-CoordAtt + BiFPN	BiFPN	**5217411**	0.571	9.3
YOLOv5s-CoordAtt+ LeakyReLU+ BiFPN	BiFPN	**5233593**	0.536	8.8
YOLOv5s-ECA + LeakyReLU + BiFPN	BiFPN	7291398	0.573	**7.9**

**Fig. 9:**
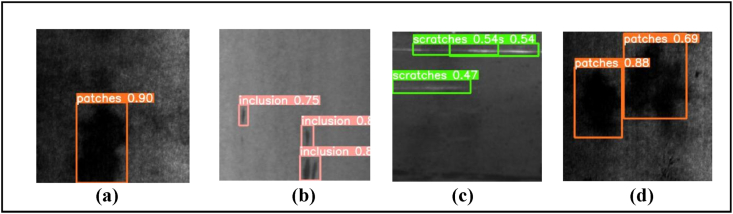
Visual detection results of YOLOv5-ECA on NEU-DET Dataset. In sequence, the pictures are: (a) Patches (Pts), (b) Inclusion (Iln), (c) Scratches(Sts), (d) Patches(Pts).

In contrast, the YOLOv5-CoordAtt, achieved a significantly higher accuracy of 0.618 compared to YOLOv5s. Inference for each model uses the optimal weights obtained during training, with a confidence threshold of 0.5. Notably, YOLOv5s-ECA exhibits shorter inference time 7.7 ms when compared to YOLOv5s. Similarly, YOLOv5s-ECA + LeakyReLU + BiFPN has inference time of 7.9 ms, YOLOv5-CoordAtt has 8.4 ms. Furthermore, the combination of CoordAtt attention mechanisms in YOLOv5s-CoordAtt, YOLOv5s-CoordAtt + LeakyReLU and YOLOv5s-CoordAtt + LeakyReLU + BiFPN effectively reduces the parameter count in comparison to YOLOv5s. Visual detection results of YOLOv5-ECA on NEU-DET Dataset are represented in Fig. 9(a)-Patches (Pts), Fig. 9(b)- Inclusion (Iln), Fig. 9(c)- Scratches(Sts) and Fig. 9(d)- Patches(Pts).

Table 8 presents the results of analyzing the GC10-DET data set using various YOLOv5 models. The YOLOv5 PANet and YOLO5vs6 models have more parameters and longer inference times than the other YOLOv5 models.

**Table 8:** Result analysis on GC10-DET dataset with different YOLOv5s Models.

Models	Neck-layer Structure	# Params.	Training mAP (%)	Inference (ms)
YOLOv5-PANet	PANet	46167513	0.642	29.9
YOLOv5s	FPN + PANet	7042489	**0.676**	8.1
YOLOv5s-Ghost	FPN + PANet	**3705393**	0.641	10.2
YOLOv5s6	FPN + PANet	12350572	0.652	10.3
YOLOv5s-ECA	FPN + PANet	7242812	0.628	10.3
YOLOv5s-CoordAtt	FPN + PANet	**5168057**	0.617	9.1
YOLOv5s-LeakyReLU	FPN + PANet	7042489	**0.667**	8.4
YOLOv5s-CoordAtt + BiFPN	BiFPN	**5233593**	0.634	9.1
YOLOv5s-CoordAtt+ LeakyReLU + BiFPN	BiFPN	**5233593**	0.653	9.0
YOLOv5s-ECA + LeakyReLU+ BiFPN	BiFPN	7321404	0.639	**7.9**

**Fig. 10:**
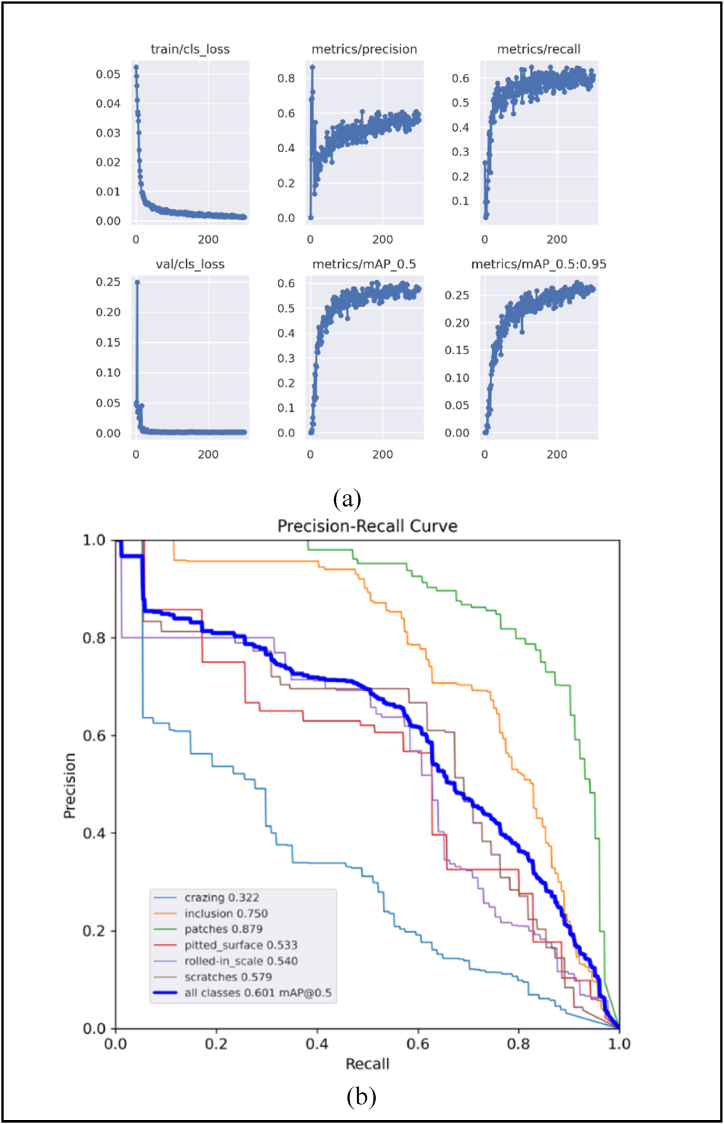
(a)Results graph (b) P-R Curve, for YOLOv5-ECA on NEU-DET Dataset.

Notably, YOLOv5s-LeakyReLU exhibits competitive performance. The inference time of YOLOv5 ECA + LeakyReLU + BiFPN is 7.9 ms lower than other models. Additionally, the use of CoordAtt attention mechanism in YOLOv5s-CoordAtt, YOLOv5s-CoordAtt + BiFPN and YOLOv5s-CoordAtt + LeakyReLU + BiFPN significantly reduces the number of parameters. Fig. 10(a) shows the results plot and Fig. 10(b) shows the P-R curve of YOLOv5-ECA on the NEU-DET dataset respectively.

**Fig. 11:**
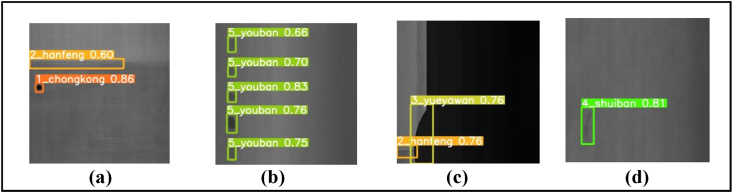
Visual detection results of YOLOv5s-ECA + LeakyReLU + BiFPN on GC10-DET Dataset. In sequence, the pictures are: (a) 1_chongkong, 2_hanfeng (punching hole, welding line), (b) 5_youban (oil spot), (c) 3_yeuyawan, 2_hanfeng (crescent gap, welding line), (d) 4_shuiban (water spot).

Visual detection results of YOLOv5s-ECA + LeakyReLU + BiFPN on GC10-DET Dataset are represented in Fig. 11(a)- 1_chongkong, 2_hanfeng (punching hole, welding line), Fig. 11(b)- 5_youban (oil spot),Fig. 11©- 3_yeuyawan, 2_hanfeng (crescent gap, welding line) and Fig. 11(d)- 4_shuiban (water spot).

**Fig. 12:**
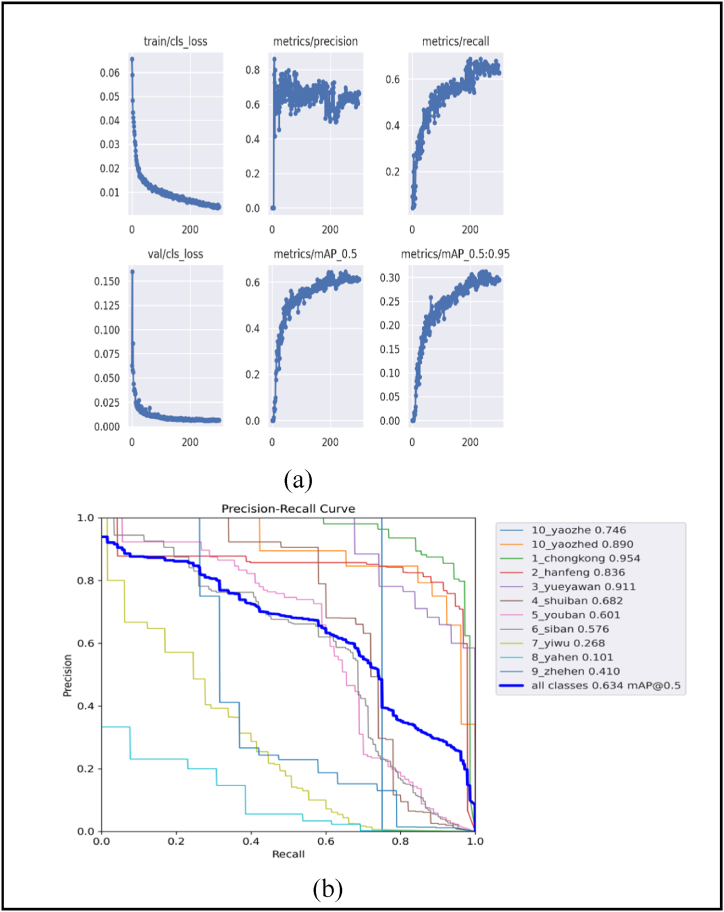
(a)Results graph (b) P-R Curve, for YOLOv5-ECA + LeakyReLU + BiFPN on GC10-DET Dataset.

Fig. 12(a) shows the results plot and Fig. 12(b) shows the P-R curve of YOLOv5-ECA + LeakyReLU + BiFPN on the GC10-DET dataset respectively. Inference time in DL refers to the duration required for a model to process new data and generate predictions.

**Fig. 13:**
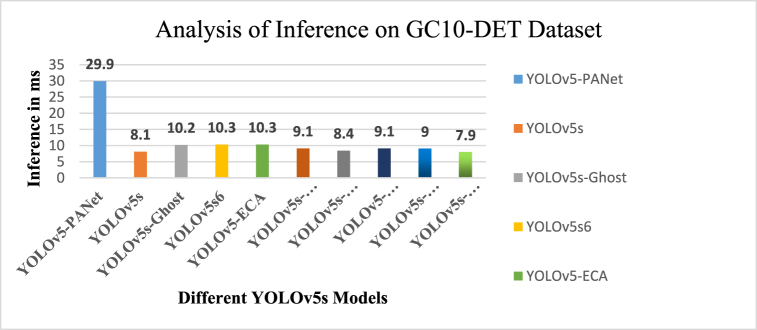
Analysis of inference on GC10-DET dataset.

Timely detection is essential for any model, regardless of the environment. In fact, most models can process new data within milliseconds. Therefore, shorter inference time indicates the model's ability to effectively predict or detect data, thereby improving performance in detection and recognition tasks. Notably, incorporating ECA reduces inference time for both datasets. Fig. 13 shows the inference analysis of the GC10-DET data set. Input size significantly affects the time complexity of most algorithms, often expressed in Big O notation. However, for DL models, according to Srivastava et al. [61], time complexity is defined as the sum of the training time of the detection model and the inference time when running the model on specific hardware.

**Fig. 14:**
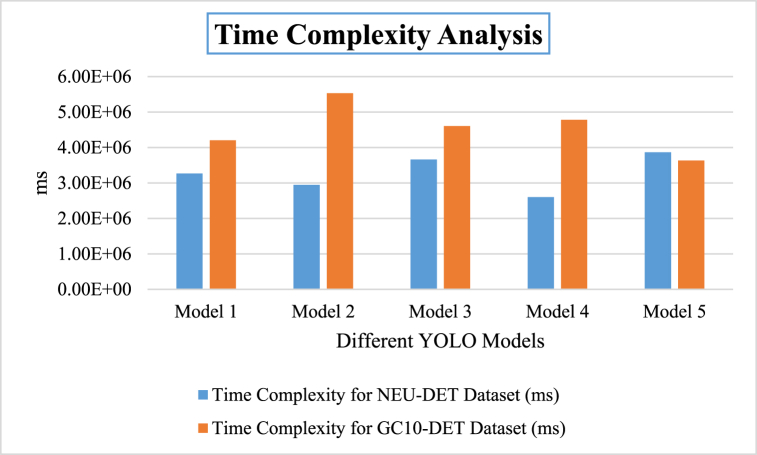
Time Complexity Analysis of different models.

Fig. 14 illustrates the time complexity of different YOLO models in our study: Model 1 represents YOLOv5s, model 2 is YOLOv5s-ECA, model 3 is YOLOv5s-CoordAtt, model 4 is YOLOv5s-CoordAtt + LeakyReLU + BiFPN and model 5 is YOLOv5s-ECA + LeakyReLU + BiFPN.

### 4.1 Validation on diverse datasets

Table 9 presents the validation results for YOLOv5 and YOLOv5s-CoordAtt on additional datasets from the ROBOFLOW framework space project, evaluating the model's generality. These datasets include PPE detection and fire smoke detection datasets. The PPE detection dataset focuses on identifying personal protective equipment (PPE) items such as gloves, masks, and helmets in manufacturing environments. It includes more than 12,870 images across 10 classes, including variations of PPE components and their absence. On the other hand, the fire smoke detection dataset aims to detect fires and smoky environments, which is essential for ensuring safety. It contains 2580 images divided into three categories: fire, smoke and others. Notably, the models demonstrate strong performance on these datasets, achieving a mean average precision (mAP) of over 80 % on the training data.

**Table 9:** Validation results of YOLOv5s and YOLOV5s-CoordAtt on various datasets.

	Datasets			
Model Name	NEU-DET (%)	GC10-DET (%)	PPE Detection (%)	Fire-Smoke Detection (%)
**YOLOv5s**	0.754	0.676	0.921	0.994
**YOLOv5s-CoordAtt**	**0.763**	0.617	0.825	0.991

Table 10 presents a comprehensive comparison of various YOLOv5 configurations used in this study with state-of-the-art models, including Mask R-CNN, SSD, RetinaNet, YOLOv6, YOLOv7, and YOLOv8 in the same experimental setup. We used smaller versions of YOLOv6, YOLOv7 and YOLOv8. All models undergo training from scratch for 100 epochs in the same experimental set up. Notably, in the NEU-DET dataset, the YOLOv5s-Ghost, YOLOv5s-ECA, and YOLOv5s-LeakyReLU outperform YOLOv6, YOLOv7, and YOLOv8. Similarly, for the GC10-DET dataset, all hybrid models except YOLOv5s-ECA and YOLOv5s-CoordAtt demonstrate higher accuracy than YOLOv6, YOLOv7, and YOLOv8. These results demonstrate the strong performance of the proposed YOLOv5 configurations, especially the hybrid models, in comparison to existing state-of-the-art models.

**Table 10:** Comparison results of different YOLOv5s models.

S.No	Name of YOLO Model	mAP@0.5 on NEU-DET Dataset (%)	mAP@0.5 on GC10-DET Dataset (%)
1.	LBP + NNC	0.380	0.478
2.	HOG + NNC	0.474	0.539
3.	HOG + SVM	0.463	0.516
4.	Mask R-CNN	0.485	0.569
5.	SSD	0.520	0.607
6.	RetinaNet	0.421	0.500
7.	YOLOv5-PANet	0.695	0.642
8.	YOLOv5s	0.754	0.676
9.	YOLOv5s-Ghost	0.692	0.641
10.	YOLOv5s6	0.743	0.652
11.	YOLOv5s-ECA	0.601	0.628
12.	YOLOv5s-CoordAtt	**0.763**	0.617
13.	YOLOv5s-LeakyReLU	0.745	0.667
14.	YOLOv5s-CoordAtt + BiFPN	0.571	0.634
15.	YOLOv5s-CoordAtt+ LeakyReLU + BiFPN	0.536	0.653
16.	YOLOv5s-ECA + LeakyReLU + BiFPN	0.573	0.639
17.	YOLOv6	0.551	0.569
18.	YOLOv7	0.48	0.60
19.	YOLOv8	0.587	0.639

## 5 Conclusion

In industrial environments, even minor errors can have severe consequences. YOLOv5 has revolutionized object and error identification and this article focuses on using YOLOv5 on detecting defects on steel strips in the NEU-DET and GC10-DET datasets. We evaluated various YOLOv5 configurations by integrating ECA, CoordAtt mechanism, LeakyReLU activation function and their combinations. Our experiments yielded promising results: on the NEU-DET dataset, YOLOv5s-ECA reduced inference time by 4 %, while on the GC10-DET dataset, the models like YOLOv5-CorrdAtt, YOLOv5s-CoordAtt + BiFPN, and YOLOv5s-CoordAtt + LeakyReLU + BiFPN achieved a 25 % parameter reduction. The highest accuracy of 76.3 % was achieved on NEU-DET dataset with YOLOv5s-CoordAtt. The tuned YOLOv5s-CoordAtt model demonstrated versatility, achieving 82.5 % mAP on PPE detection and 99.1 % mAP on fire and smoke detection datasets. Furthermore, comparative analysis with recent YOLO versions and other models like Mask R-CNN and SSD showed that integrating attention mechanisms and activation functions improves detection and recognition performance. In further research evaluation and directions, we aim to build a dataset to validate our model's effectiveness. We plan to improve accuracy, minimize false positives, optimize non-rigid detection effectiveness, fine-tune the network model structure, explore advanced learning techniques like transfer learning and meta-learning, enhance image data fusion using GAN and develop tailor-made models with improved real-world data.

## CRediT authorship contribution statement

**G. Deepti Raj:** Writing – original draft, Visualization, Validation, Software, Resources, Methodology, Investigation, Formal analysis, Data curation, Conceptualization. **B. Prabadevi:** Writing – review & editing, Supervision, Resources, Project administration, Funding acquisition, Formal analysis, Conceptualization.

## Informed consent statement

Informed consent was not required as the study did not involve human participants.

## Data availability statement

The datasets analyzed during the current study are available in the Kaggle Website and the links for the datasets are given below.

1. https://www.kaggle.com/datasets/kaustubhdikshit/neu-surface-defect-database
2. https://www.kaggle.com/datasets/alex000kim/gc10det

## Ethics approval

Ethical approval was not required as the study did not involve human participants.

## Funding statement

Not Applicable.

## Declaration of competing interest

The authors declare that they have no known competing financial interests or personal relationships that could have appeared to influence the work reported in this paper.
